# Hepatitis D: challenges in the estimation of true prevalence and laboratory diagnosis

**DOI:** 10.1186/s13099-021-00462-0

**Published:** 2021-10-30

**Authors:** Lin-Yuan Chen, Xiao-Yu Pang, Hemant Goyal, Rui-Xia Yang, Hua-Guo Xu

**Affiliations:** 1 Department of Laboratory Medicine, the First Affiliated Hospital of Nanjing Medical University, Nanjing, China; 2 Department of Internal Medicine Macon, Mercer University School of Medicine, Georgia, USA

**Keywords:** Hepatitis delta virus, Hepatitis D, Hepatitis B, Prevalence, Diagnostics

## Abstract

Hepatitis delta virus (HDV) is a defective single negative chain RNA virus, as its envelope protein synthesis is dependent on hepatitis B virus (HBV). Studies have consistently shown that coinfection of HBV and HDV is the most serious form of viral hepatitis, with accelerated progression to liver cirrhosis and hepatocellular carcinoma. About 74 million of HBV surface antigen (HBsAg) positive patients worldwide are also co-infected with HDV. Besides, patients with intravenous drug use and high-risk sexual behavior are at higher risk of HDV infection. Therapeutic schedules for HDV are limited, and relapse of HDV has been observed after treatment with pegylated interferon alpha. To reduce the transmission of HDV, all people infected with HBV should be screened for HDV. At present, several serological and molecular detection methods are widely used in the diagnosis of HDV. However, due to the lack of international standards diagnostic results from different laboratories are often not comparable. Therefore, the true prevalence of HDV is still unclear. In this manuscript, we have analyzed various factors influencing the estimation of HDV prevalence. We have also discussed about the advantages and disadvantages of currently available HDV laboratory diagnostic methods, in order to provide some ideas for improving the detection of HDV.

## Introduction

**Fig. 1:**
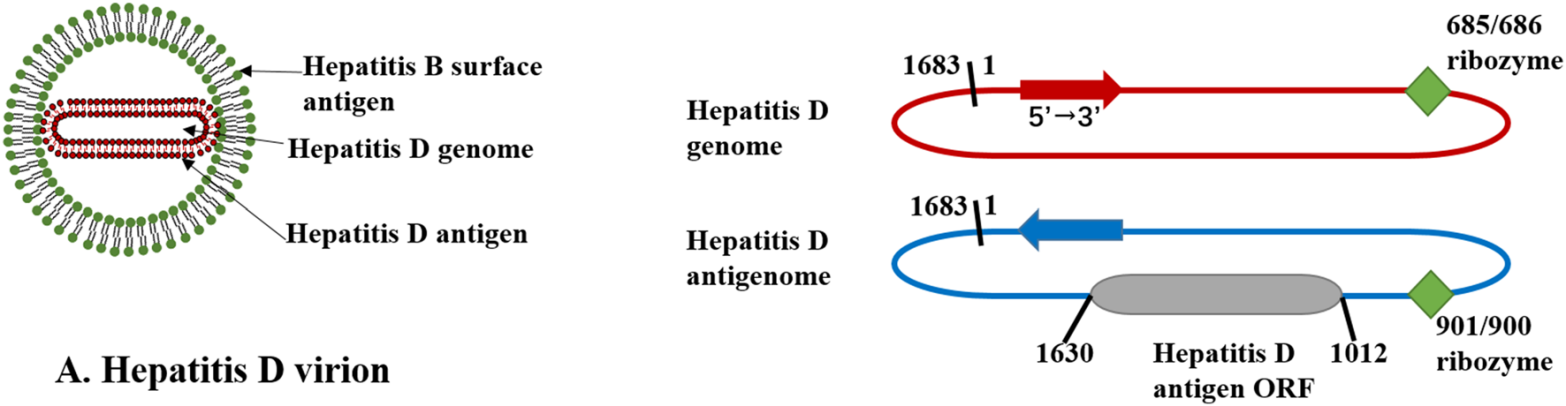
HDV virion and nucleic acid structure diagram. **A** HDV virion has a ribonucleoprotein (RNP) complex inside and an HBV derived envelope outside. The RNP consists of the HDV genome and two isoforms of hepatitis D antigen (HDAg), L-HDAg and S-HDAg. **B** Structure of HDV RNA. Both genome and antigenome form an unbranched rod-like structure of 1700 bp containing a self-cleaving ribozyme (green boxs). The gray box represents HDAg ORF. Arrows on the RNA strands indicate the 5′–3′ direction. *HDV* hepatitis D virus; *L-HDAg* large hepatitis D antigen; *S-HDAg* small hepatitis D antigen

**Fig. 2:**
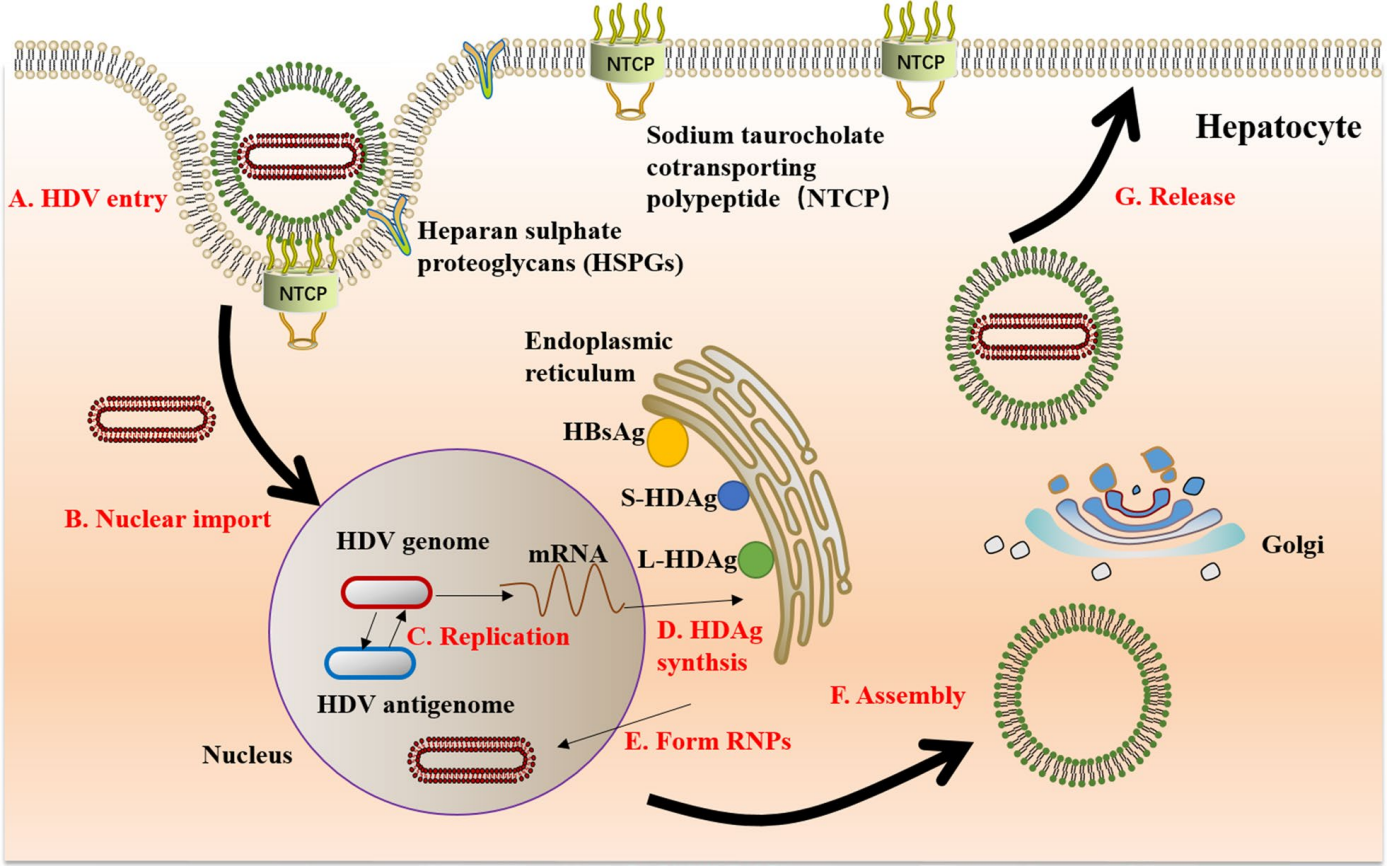
Schematic representation of HDV life cycle. **A** HDV virion enters the hepatocyte via HSPGs and NTCP. **B** The virion loses its envelope and the RNP is imported into the nucleus of the cell. **C** Within the nucleolus, HDV RNA is replicated using a double rolling circle amplification to form the antigenomic RNA and more genomic RNA. **D** The mRNA is exported to the cytoplasm where it is translated at the endoplasmic reticulum (ER) to form HDAg. **E** HDAg return to the nucleus where the S-HDAg isoform promotes further genome replication. S-HDAg and L-HDAg bind to new transcripts of genomic RNA to form new RNPs. **F** RNPs are exported to the cytoplasm where L-HDAg facilitates association with HBsAg in the ER to assemble new virus particles. **G** They are then released out of the hepatocyte via the Golgi to infect neighboring cells. *HDV* hepatitis D virus; *L-HDAg* large hepatitis D antigen; *S-HDAg* small hepatitis D antigen; *HBsAg* hepatitis B surface antigen; *RNP* ribonucleoprotein; *mRNA* messenger RNA; *NTCP* sodium taurocholate cotransporting polypeptide; *HSPGs* heparan sulphate proteoglycans

Hepatitis Delta virus (HDV) is a blood-borne pathogen that is dependent on the hepatitis B virus (HBV) for its life cycle. HDV was first identified in HBV-infected patients with severe hepatitis by Rizzetto et al. in 1977 [1]. Initially, HDV was thought to be a new HBV antigen, which was named delta antigen. However, later it was found to have a unique structural component of infectious pathogens and associated with HBV surface antigen (HBsAg) in experimentation with chimpanzees [2]. Wang et al. cloned and sequenced the genome, and it turned out to be a unique RNA virus [3]. HDV is the only member of the delta virus genus, originating from plant viroids or cellular circular ribonucleic acids [4]. HDV is currently the smallest virus capable of infecting humans [5], with a particle diameter of 36 nm and a genome of only about 1.7 kb which can encode two forms of hepatitis D antigen (HDAg), namely the small HDAg (S-HDAg) and the large HDAg (L-HDAg) [6]. S-HDAg has 19–20 fewer amino acids than L-HDAg, but they come from the common 0.8 kb mRNA [7]. S-HDAg is necessary to initiate and maintain HDV RNA replication [8], while L-HDAg inhibits HDV replication [9], but is crucial for the assembly of virions [10]. The HDAg binds to the HDV genome to form a ribonucleoprotein (RNP) complex. The HDV genome contains two self-cleaving motifs called ribozyme regions [11], located upstream to the region coding for HDAg (Fig. 1). HDV replicates only in liver nuclei, but the virion is defective. As an envelope protein, HBsAg allows HDV to enter hepatocytes. HDV and HBV first bind to heparan sulphate proteoglycans (HSPGs) on the surface of hepatocytes [12] and then enter host cells via sodium taurocholate cotransporting polypeptide (NTCP) [13]. After membrane fusion, RNP is released and further transported to the nucleus, where the mRNA encoding S-HDAg is transcribed and translated. The entering genomic RNA serves as a template for the first roll-loop amplification. The resulting antigenomic RNA polymers are cleaved by self-cleaving RNA sequences (namely ribozyme) and linked into circular monomers [14]. Using antigenomic RNA as a template, the HDV genomic RNA polymers are synthesized and further cleaved to form monomers. Editing of HDV antigenomic RNA by cellular adenosine deaminase 1 (ADAR 1) allows L-HDAg transcription and translation [15]. S-HDAg and L-HDAg are transported into the nucleus to regulate viral replication or to bind to HDV RNA to form RNP. RNP containing HDV genomic RNA can be exported to the cytoplasm and encapsulated into the HBV envelope through the interaction between L-HDAg and HBsAg. HDV virions are released through the ER-Golgi secretory pathway. (Fig. 2) In addition to de novo infection dependent on the HBV envelope, HDV can directly transfer replicable HDV RNA between cells during mitosis [16]. There are currently eight HDV genotypes, but HDV has a high degree of genetic heterogeneity [17]. The differences between isolates of the same genotype were less than 16%, while the degree of separation between genotypes was as high as 40% [18]. The clinical HDV infection can occur in two manners; 1. Coinfection: when HDV and HBV simultaneously infect the host causing acute viral hepatitis, and 2. Superinfection: HDV infection occurring in HBsAg carriers or chronic HBV infected individuals leads to chronic viral hepatitis [19]. Chronic hepatitis D (CHD) is the most serious type of viral hepatitis in humans [20]. Patients with HDV are more likely to develop liver cirrhosis [21], liver decompensation [22], and are at increased risk of hepatocellular carcinoma (HCC) [23] than other chronic viral hepatitis. Although the underlying mechanisms of HDV infection are not fully understood, HDV load (HDVL) can influence the course of liver disease to some extent [24]. Moreover, there is no available effective treatment for HDV at present [25]. The commonly used pegylated interferon alpha (IFN-α) inhibits HDV replication in about 25–30% of patients, but this therapy has side effects and is also associated with late recurrence [26]. The quantification of viral nucleic acid is very important for monitoring the occurrence and development of disease and evaluating the therapeutic effect. Therefore, it is paramount to develop an economical, sensitive and specific international standardized test for the detection of HDV RNA.

## Prevalence

Epidemiological estimates have suggested that about 5% of patients with hepatitis B were also co-infected with HDV globally, which equated to about 15 million individuals [27]. However, a recent meta-analysis found that the seroprevalence of HDV in mixed population was about 10.07% between 1977 and 2018, meaning that about 74 million people worldwide were infected with HDV [28]. At the same time, Miao et al. analyzed articles published from 1980 to 2019 and suggested that the total prevalence of HDV among HBV carriers was 13.02%, corresponding to 48–60 million individuals worldwide [29]. In addition, another study, which searched and analyzed articles from 1st January 1998 to 28th January 2019, Q-MAC suggested that the prevalence of HDV in all HBsAg positive patients was about 4.5%, or about 12 million people worldwide were infected with HDV [30]. These differences in the variation in HDV prevalence estimates could be due to several reasons. First of all, the criteria for inclusion and exclusion of the literature varied significantly producing different prevalence. Most of the published literature used the presence of HDV antibodies as the diagnostic criteria, but anti-HDV positivity may not differentiate between an existing or a past infection [31]. Second, the prevalence and the genotype distribution of HDV vary widely in different countries and regions. The prevalence of HDV is high in Central Asia, Eastern Europe, West Africa and the Amazon basin [31]. However, due to a limited number of studies in some countries, the estimated value may not represent the true prevalence in those countries [32]. Besides, patients with intravenous drug use (IVDU), high-risk sexual behavior (HRSB), human immunodeficiency virus (HIV) or hepatitis C virus (HCV) infections are at higher risk of HDV infection [32]. Thus, the current prevalence of HDV is still controversial. High-quality global studies with consistent inclusion and exclusion criteria are needed to estimate the true prevalence of HDV.

## Current laboratory diagnosis

Traditionally, the diagnosis of a viral disease has relied on culture-based methods that directly detect the intact virus or its components (proteins or nucleic acids) or serological tests that indirectly detect the antigens or antibodies of the viruses [33]. Nowadays, polymerase chain reaction (PCR)-based methods are widely used in virus diagnosis because of their high specificity, sensitivity and ease of operation. These tests can rapidly amplify certain parts of the genome necessary for virus identification. Current methods for detecting HDV RNA are described below.

### Serological diagnosis

**Fig. 3:**
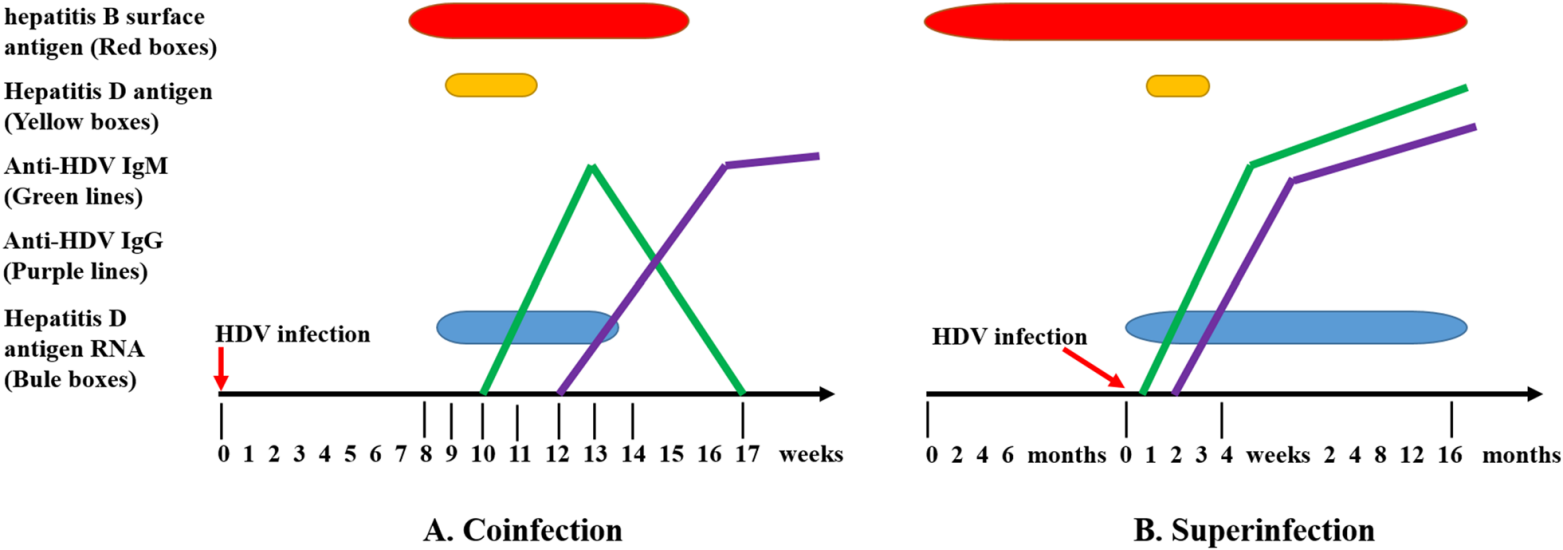
Serological patterns of HDV infection. **A** Coinfection is the simultaneous acute infection of HBV and HDV in a susceptible individual. Serum HDAg is detectable only transiently in blood specimens collected early at the onset of HDV, before the rising of antibodies. Anti-HDV IgM response is rapid and weak suggesting a resolution of infection. Anti-HDV IgG levels increased rapidly and persisted. **B** Superinfection is an HDV infection in an individual chronically infected with HBV. This pattern of infection has two components, the acute stage and the chronic stage. The acute phase is characterized by very high levels of HDV viremia and HDAg antigen in serum/liver. In the chronic period, HDV RNA, anti-HDV IgM and anti-HDV IgG persist. HDAg can be detected by liver biopsy. *HDV* hepatitis D virus; *HBV* hepatitis B virus; *HDAg* hepatitis D antigen; *HBsAg* hepatitis B surface antigen; *Anti-HDV* *IgM* immunoglobulin M antibody to the HDAg; *Anti-HDV* *IgG* immunoglobulin G antibody to the HDAg

Not long after discovery of HDV, antibody tests were developed and marketed as serological markers of HDV infection [34]. Currently, antibody testing is often used as a primary screening test for HDV infection, with detection of antibody to the HDAg (anti-HDV) by enzyme-linked immunosorbent assay (ELISA) or radioimmunoassay (RIA). The immunoglobulin M (IgM) anti-HDV is detectable within 2–3 weeks of symptom onset and disappears 2 months after acute infection [35]. However, anti-HDV IgM is also elevated in patients with chronic HDV during disease flares [36], so detection of anti-HDV IgM cannot definitively distinguish between acute and chronic HDV infections. The anti-HDV IgG is positive in patients with acute remission of HDV infection and chronic HDV infection, and persists for a long time after virus clearance, making it difficult to distinguish between present and previous HDV infections (Fig. 3). Chen et al. have used protein microarray technology to develop a Q-MAC assay for sensitive quantitative fluorescent detection of anti-HDV IgG from patient sera [37]. Anti-HDV IgG has been quantitatively detected by quantitative microarray antibody capture (Q-MAC) in Mongolia, the United States and the Gambia [37–39]. Although there is a certain correlation between the fluorescence intensity of Q-MAC and HDV RNA, the relationship could not be determined for co-infection with hepatitis C virus (HCV), also known as triple hepatitis virus infection [38, 40]. HDAg is expressed in liver cell nuclei and can be detected by immunochemistry. Detection of serum HDAg is limited and detectable in the first 2 weeks of acute infection [41]. Intrahepatic HDAg can be detected by immunohistochemistry in liver biopsy samples, but this method is not commonly used.

### Molecular diagnosis

The HDV nucleic acid quantitative analysis can be used to both diagnose and monitor the treatment outcomes. The routine PCR cannot be used to detect HDVL as the HDV genome is made of RNA, not DNA [42]. Real-time quantitative reverse transcript PCR (RT-qPCR) is to add a signal system based on traditional PCR to achieve the purpose of real-time quantitative detection of samples. According to the different signal, groups can be divided into fluorescent dye method and probe method. Currently, there are a number of internal methods and commercial kits available for quantitative determination of HDV RNA (Table 1). These techniques are designed to target the sequences coding for the conserved regions of the HDAg or the ribozyme domain. HDV genome has a robust secondary structure, which can be destroyed by adding heat shock treatment prior to the reverse transcriptional step [43–47], thus improving reverse transcriptional efficiency. The use of one-step RT-qPCR reduces the risk of contamination during reverse transcription and amplification [47–50]. The standard used in most published studies is HDV plasmid DNA [43–46, 51–53], but this does not assess reverse transcriptional steps. Other studies have used RNA synthesized in vitro as the standard [47, 48, 50, 54, 55]. Although reverse transcription steps can be evaluated, the synthesized RNA does not have a strong secondary structure, and the secondary structure of the HDV genome also affects the quantitative viral load. In the sixty-fourth report of the World Health Organization (WHO) Expert Committee on Biological Standardization, a sample with a high HDV titer (code 7657/12) was tested in 19 laboratories around the world with a lower limit of 5,75,000 IU/mL, based on the averages reported by different assay methods [56, 57]. Although the WHO has proposed an international standard for HDV RNA tests, but it still needs internal control, which should be co-purified and co-amplified with the nucleic acid being tested. Competitive RNA internal controls have been devised that use the same primers to detect both the control and the target virus [58]. However, detection accuracy decreases when the target viral load is low. Noncompetitive internal controls have also been described, using separate primers to detect controls and samples. Some housekeeping genes such as β-actin and 18S rRNA are used for internal controls [59]. Unfortunately, the concentrations of these RNAs varied greatly among clinical samples. After Karataylı et al. put forward the free circulating nucleic acids in plasma and serum (CNAPS) that is Glyceraldehyde-3-phosphate dehydrogenase (GAPDH) as internal reference [60]. However, the authors found that the nature of freely circulating GAPDH in serum was mostly in DNA nature and a small portion of GAPDH in RNA nature by RNAse or DNAse digestion in a number of HDV and HBV patients. Therefore, GAPDH is not an ideal internal control for detecting RNA viruses [61]. Ferns et al. used heterogenous RNA that is Brome Mosaic virus (BMV) RNA, as an internal control. 10 fg BMV RNA was added to each sample and the RNA was extracted. RT-qPCR assay was performed using specific primers/probes of HDV and BMV. Full-length genomic HDV RNA was used as calibration criteria. However, when the HDV RNA concentrations below 7000 copies/ml, the detection became less accurate. Moreover, the use of more than 10 fg BMV RNA per sample would reduce the sensitivity of HDV detection [48]. Although a number of commercial tests have been developed for HDV RNA quantification [49, 50, 60–62], they underestimate or fail to quantify HDVL [63]. The international external quality assessment for HDV RNA quantification in plasma was first conducted in 2016. A comprehensive analysis of 28 laboratories in 17 countries worldwide showed that the results were highly heterogeneous due to the differences in the detection techniques and procedures, and the different target areas for which the primers or probes were designed [64]. Therefore, it is necessary to establish an international detection system for the quantification of HDV RNA. In addition, Wang et al. detected HDV genotype 1 by reverse transcription-loop-mediated isothermal amplification (RT-LAMP) [65]. LAMP employs DNA polymerase, a set of four specially designed primers that can identify a total of six different sequences in the target DNA [66]. RT-LAMP is similar to LAMP, but the template used is RNA instead of DNA. Compared with conventional PCR, LAMP is simple to operate, only needs to be incubated under isothermal conditions, and can quickly and efficiently amplify the target sequence in a short time. However, as the LAMP technology can identify white precipitate with naked-eye, its detection results cannot be quantified accurately [67]. Different genotypes of HDV have an impact on the clinical outcome of infection, with genotype 1 being the most common genotype and having variable pathogenicity [68]. The genotypes of HDV can be determined by phylogenetic analysis, providing evidence for more accurate diagnosis of HDV in clinic [69].

**Table 1:** Summary of qRT-PCR for HDV

Technique	Target site	Linear ranges	Advantage	Disadvantage	References
SYBR Green I qRT-PCR	A highly conserved sequence within the HDAg-coding region	1–10^6^ copies	A sensitive method for the detection of HDV RNA at levels as low as a single copy	HDV is prone to mutations that can cause primer mismatch	[54]
TaqMan probe qRT-PCR	The ribozyme region of the genome	10–10^7^ copies	Avoid post-PCR handing that can be a source of DNA carryover	For HDV-3 isolates, the forward primer will mismatch	[51]
Sybr green I qRT-PCR	The HDAg coding region	10^3^–10^9^ copies	Detect HDV genomic RNA, antigenomic RNA, and HDAg-encoding mRNA in an HDV cDNA-free RNA transfection system	The primers are designed according to HDV-1 and HDV-2	[55]
Hybridization probe qRT-PCR	Highly conserved regions	2 × 10^3^–10^8^ copies/ml	This approach facilitates a subsequent typing based on melting curve analysis	Only quantitative detection of HDV-1 and HDV-3	[43]
Cobas TaqMan-based in-house PCR	A conserved region that encodes the HDAg	3 × 10^2^–1.5 × 10^8^ copies/ml	This platform provides the automated extraction system, a minimal risk of contamination and a high degree of reproducibility	The DNA clone does not accurately monitor the expansion of RNA viruses	[52]
Hybridization probe qRT-PCR	A highly conserved region	10^3^–10^8^ copies/ml	High sensitivity and a wide dynamic range	A cloned cDNA does not permit assessment of the RT step	[44]
In-house qRT-PCR	The highly conserved ribozyme and s-HDAg regions	10^1^–10^7^ copies/reaction	This technology offers more options for primer design	This has proven challenging due to the high genetic diversity of the HDV genome	[45]
TaqMan probe qRT-PCR	The highly conserved ribozyme region of the HDV genome	7 × 10^2^–7 × 10^6^ copies/ml	A BMV RNA internal control which effectively monitors all stages of the assay	Using more than 10 fg BMV RNA per sample can reduce HDV assay sensitivity	[48]
Quantitect Virus kit	The conserved parts of the gene encoding the delta antigen	2.7 × 10^1^–2.7 × 10^11^ copies/reaction	A one-step qRT-PCR can be automated for the accurate quantification of HDV in Europe in the presence of an encapsulated heterologous RNA used as an internal control	HDV-1 cannot be completely detected	[49]
Molecular beacon qRT-PCR	The ribozyme domain of HDV genome	13–13 × 10^10^ copies/ml	High sensitivity, reproducibility and wide detection range	A synthetic DNA has been used as standard	[46]
AgPath-ID One-Step kit	The ribozyme region of HDV genome	7.5 × 10^2^–7.5 × 10^8^ copies/ml	The positive control transcript controls for every step of the qRT-PCR	The clinical samples tested do not contain all HDV genotypes	[50]
One step EZ RT PCR kit	The conserved regions of HDAg	10^2^–10^11^ copies/ml	A new protocol having armored RNA as external standard and GAPDH as the intrinsic internal control in single tube format	The extracted samples contain abundant DNA-natured GAPDH, while only a small amount of RNA-natured GAPDH	[60, 61]
The Eurobioplex HDV kit	HDAg coding region	2.75–8.5 log IU/ml	The kit exhibited good clinical agreement with the French National Reference Laboratory (FNRL) assay and detected all HDV genotypes	Tendency to underestimate HDV-5 to -8	[62]

## Diagnosis challenges

Several issues exist for the correct diagnosis of HDV, ultimately affecting its true prevalence. Ideally, HDV diagnosis should be made with the identification of both HDV antibody and RNA, to distinguish between the chronic and previous infections, and to monitor treatment response [70]. Most antibody tests are ELISA kits, but the accuracy of internal and commercial quantitative analysis of HDV RNA varies significantly, and sometimes HDV RNA cannot be detected in HDV RNA-positive samples [64]. Bremer et al. found that automated nucleic acid isolation led to viral load underestimation by comparing manual and four automated HDV RNA extraction methods (AmpliPrep, MagNA Pure, QlAcube QBK and QlAcube VRK) [24]. Moreover, the secondary structure and different HDV genotypes should be considered when designing primers and probes [64]. Besides, the choice of quality control products will also affect the accurate quantification of HDV RNA. The ideal viral nucleic acid quality control products can not only be used for the quality control of nucleic acid detection process, but also an important material basis for the evaluation of detection procedures and the comparison of results between different laboratories [71]. At present, the quality control products of RNA viruses are mostly bare RNA fragments or whole virus particles. Bare RNA fragments, as quality controls, are easily degraded by RNase in the environment and cannot effectively react with to the nucleic acid extraction process [72]. Traditional RNA preservatives such as alcohols and guanidine can lead to RNA degradation [73]. RNAlater is a good RNA stabilizer, but has different effects on enveloped and non-enveloped viruses. For example, at room temperature, after long term storage of RNAlater, the cellular infectivity of non-enveloped enterovirus remained high, but the cellular infectivity of enveloped vesicular stomatitis virus decreased [74]. The whole virus particles, especially the inactivated virus particles, have safety risks as quality control materials. Although inactivated drugs can reduce infectivity, there is still a risk of incomplete inactivation, and virus inactivation reagents (such as formaldehyde) will destroy viral nucleic acid, affecting the extraction of its nucleic acid [75]. The ideal RNA quality control products should be stored for a long time without degradation or loss of copy number [76]. Armored RNA technology can overcome RNA instability and has been widely used in the research of RNA viral nucleic acid quality control products. Pasloske et al. invented armored RNA by building the quality controls for HIV [77]. The principle of this technology is the use of genetic engineering methods to clone a sequence containing phage coat protein gene and target fragment into the expression vector. The vector transcribed the cloned fragment into recombinant RNA and assembled it into spherical RNA–protein complex, namely RNA virus-like particles, using the shell protein synthesized from the shell protein gene on the vector [78], thus preventing the RNA degradation.

## Conclusion

Although the true prevalence of HDV remains unclear, multiple epidemiological studies, have revealed that the global burden of HDV is higher than previously estimated. Rapid laboratory diagnosis of HDV infection is of significant importance for identifying, monitoring, and controlling the disease spread. At present, the sensitivity of different HDV RNA detection methods varies substantially, and there is a lack of international unified detection standard for comparable results between different laboratories. In addition, insufficient healthcare access in low-and middle-income countries often leads to underdiagnoses of the disease hindering the early diagnosis and effective treatment. The RT-qPCR assay has been developed. However, the development of new detection methods is still needed that not only have high sensitivity and specificity but also can monitor a series of processes such as HDV RNA extraction, reverse transcription, and quantification, thereby reducing false-negative rates, identifying HDV patients earlier, and controlling HDV transmission in a timely manner. In addition, standardized procedures need to be established, such as sample type, extraction method, primer/probe designs, protocols, instrumentation, and reporting units. Therefore, the future goal is to standardize these tests for the accurate detection of HDV. It is possible that further innovations, such as mass spectrometry and real-time biosensor platforms, will detect HDV RNA or its components and monitor its occurrence.

## Data Availability

Not applicable.
